# Barriers and Facilitators for the Implementation of an Online Portal in Hospital Mental Health Care: Implementation Study

**DOI:** 10.2196/82450

**Published:** 2026-05-19

**Authors:** Isabelle Reinhardt, Laura I Thomas, Lena Oster, Laura Kuhlmann, Jürgen Zielasek, Dieter F Braus, Michael Franz, Florian G Metzger, Euphrosyne Gouzoulis-Mayfrank

**Affiliations:** 1 LVR-Institute for Research and Education, Research Branch, Wilhelm-Griesinger Str. 23, Cologne, 51109, Germany, 49 2218993287; 2 Society for Digital Health (Gemeinnützige Gesellschaft für digitale Gesundheit GDG mbH), Kassel, Germany; 3 Medical Faculty, Heinrich Heine University Düsseldorf, Düsseldorf, Germany; 4 Vitos Clinic Rheingau, Eltville, Germany; 5 Vitos Clinic for Psychiatry and Psychotherapy Giessen, Giessen, Germany; 6 Justus Liebig University Giessen, Giessen, Germany; 7 Vitos Hospital for Psychiatry and Psychotherapy Haina, Haina, Germany; 8 University Hospital Tübingen, Department of Psychiatry and Psychotherapy and Geriatric Center, Tübingen, Germany; 9 LVR Clinic Cologne, Cologne, Germany

**Keywords:** digital platform, electronic patient portal, hospital mental health care, barriers and facilitators, implementation research

## Abstract

**Background:**

In recent years, digital patient portals have become an increasingly common feature of care in various medical fields. Despite growing scientific evidence of their effectiveness and the benefits they offer to patients and caregivers, their implementation, especially in hospital mental health settings, lags behind expectations.

**Objective:**

The study aimed to identify the barriers and facilitators to implementing a patient portal in a public mental health hospital setting in Germany. Moreover, it aimed to develop recommendations for implementing a patient portal.

**Methods:**

Three psychiatric clinics in the early stages of implementing an online portal for patients participated in this implementation study. We assessed objective usage data (log data from the patient portal) and performed qualitative interviews with professionals and questionnaire surveys with both patients and professionals. We combined the results to develop generic recommendations for the implementation of patient portals in a mental hospital setting using a 2-stage Delphi method with a group of professionals and patients.

**Results:**

Portal log data from 71 patients indicated variation in the use of the portal functions. On average, users logged in 9.5 (SD 14.9) times (median 4, IQR 2-7 times). The variability in the number of logins per patient, ranging from 1 to 72, indicated a high variance in the frequency of use. On average, the portal was used for 47 (SD 59) days (median 27, IQR 2-62 days). Questionnaire data from 27 patients showed satisfaction with the portal and elucidated perceived barriers to usage. Qualitative interview data from 15 professionals revealed patient-related, professional-related, organizational, structural, and technical facilitators and barriers to the implementation process. We developed 10 actionable recommendations for the implementation of digital patient portals in psychiatric hospitals, which were rated by an expert group on different dimensions.

**Conclusions:**

To our knowledge, this is the first implementation study in a German mental health hospital setting that provides experience-based recommendations for advancing the implementation of digital patient portals in hospital mental health care. The next steps will include the analysis of a larger number of users and functions, which will help to specify recommendations for different target groups and settings.

## Introduction

Electronic patient portals have become increasingly common in health care. They integrate multiple digital functions, such as appointment scheduling, access to medical information, assessments, and communication, and may improve patient-provider communication, patient engagement, self-management, and efficiency in hospital settings [1-4]. In psychiatric hospitals, electronic mental (e-mental) health apps are gaining relevance, with blended interventions showing small but significant positive effects on clinical outcomes [5]. However, patient portals remain less common in mental health care. A scoping review identified only 31 studies evaluating their use, with limited evidence on effectiveness and messaging as the most frequently implemented function [6]. A Canadian study and a German study indicate that patient portals in mental health care support timely information exchange, strengthen clinician-patient relationships beyond appointments, and improve care delivery [7,8]. Their implementation has been associated with increased patient activation, appointment adherence, autonomy, and improved access to digital communication and therapies [6].

The use of patient portals is influenced by multiple factors. A review of 120 studies found that patient engagement depends on personal characteristics such as age, ethnicity, education, health literacy, and health status, as well as provider endorsement and ease of use [9]. Organizational support, positive attitudes toward reducing paperwork, clinician acceptance, and guidance from clinical supervisors further facilitate portal implementation and use [10,11].

Key barriers include limited resources, financial constraints, and privacy and security concerns [10]. Additional challenges reported in other medical fields involve insufficient staff training and technical, organizational, and administrative factors [12,13]. In Germany, few studies have evaluated psychiatric hospital apps, and many clinicians lack practical experience with online mental health care [12,14-16].

In sum, there is a significant gap between the development of effective internet-based apps for mental disorders and their provision and use in routine care. Implementation research aims to close this gap [17]. The process of adapting and implementing e-mental health apps should involve clinicians, users, and administrative staff [18]. European policy recommendations have been formulated to foster the implementation of digital mental health care [19]. In order to move beyond identifying barriers and facilitators and explicitly develop or propose methods for deriving implementation recommendations or strategies from them, studies used implementation frameworks for determinant identification, organization, and mapping to strategy types or combined expert consensus, stakeholder input, and, where feasible, empirical feedback or data-driven models to prioritize strategies for specific determinant profiles [20].

Our previous review of studies indicated that specific measures are needed to implement electronic patient portals in inpatient psychiatric services to support adoption [18]. An earlier study assessed professionals’ attitudes toward such portals via an online survey [21], showing high acceptance but also noting technical, structural, organizational, and staffing barriers, along with perceived benefits [21]. This study evaluated the initial implementation of an online patient portal in a German public hospital, combining objective usage data, patient-reported data, and qualitative interviews with professionals in a mixed methods approach to gain deeper insights into different perspectives. The findings informed recommendations for advancing patient portal implementation in psychiatric hospital care.

## Methods

### Online Portal and Setting

The electronic patient portal Curamenta is a professional service provided by 7 major public mental health service providers in 5 federal states of Germany (as of 2023). These providers operate 45 psychiatric hospitals, which together offer care to around 650,000 people per year on an inpatient, day-care, or outpatient basis. Patients gain access to the portal via their treating inpatient physician or therapist and are invited to use it during their inpatient or day-patient stay. At the time of the study, the portal offered the following functions (Table 1).

**Table 1.** Functions of the platform (as of the study conducted in 2025).

Function	Description
Patient diary	Patients may create (write) a personal diary. They can share the diary or excerpts with their treating physician or therapist.
Messenger	The messenger function can be used for text messages. Only the treating physician or therapist is able to open and close the channel.
Notes	Patients can add free-text.
Material pool	The material pool contains worksheets, questionnaires, and other documents. These can be provided by the treating physician and other members of the treatment team (“assign”). Patients may work on assigned documents and send back the filled-out forms afterward to their therapist (“share”).
Appointments or weekly schedule	Calendar and appointment function: Patients can view their weekly time schedule. Changes or cancelations can be entered by the treating physician.

Patients accessed Curamenta via their smartphone, while professionals accessed Curamenta using their work-PCs at their workplace. At the time of the study, the portal did not provide access to any medical records, laboratory results, or clinical visit notes. Although the portal had been technically implemented in all clinics, the number of users varied across the different sites.

The implementation process of the portal is supported by an umbrella organization (GDG, Society for Digital Health), which is a nonprofit organization connecting partners at the provider level. All regional Curamenta service providers are GDG associates. At the time of the study (June 2024-May 2025), the implementation process was still in its early stages. Not all providers had started training staff or implementing the portal into routine care. Three clinics of one of the providers (Vitos in the federal state of Hesse, Germany) were chosen for this initial study, as they had trained staff and were already using the portal since 2023. The study was conducted by the research institute of one of the other 4 providers (the Rhineland Regional Council Institute for Research and Education [LVR-Institut für Forschung und Bildung]) in collaboration with the GDG and a large, nationwide patient advocacy organization (Federal Network for Mental Health Self-Help [Bundesarbeitsgemeinschaft Selbsthilfe Seelische Gesundheit]).

### Study Design

**Figure 1.**
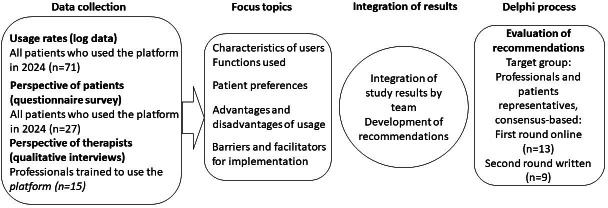
Study design.

Funding was obtained from the German Federal Ministry of Health (Bundesministerium für Gesundheit; 2524FEP20A). Three work packages were dedicated to data collection. This included the analysis of objective quantitative data (secondary data, usage data) and primary quantitative as well as qualitative subjective data (eg, usefulness, perceived barriers, and facilitators of implementation) from the perspective of the main user groups involved (patients and professionals). Implementation recommendations were derived by the project team from the data analysis results and evaluated by a selected group of professionals and psychiatric patient representatives as part of a 2-stage Delphi process (Figure 1). This study was reported according to the GRAMMS (Good Reporting of a Mixed Methods Study) checklist (Checklist 1) [22]. The COREQ (Consolidated Criteria for Reporting Qualitative Research) checklist (Checklist 2) [23] was used for quality assurance in the qualitative part.

### Portal User Log Data

The responsible IT company (Xtension) provided usage data in the form of comma-separated values files, which we analyzed as server log data. We analyzed all available data from patients who actively used the Curamenta patient portal (ie, logged in at least once) in the 3 participating clinics in 2024. Data for all available variables (eg, gender, age, functions used) were obtained from the portal (in October 2024 and January 2025), anonymized by the IT company, and transmitted to the Institute for Research and Education of the Rhineland Regional Council Institute for analysis (Multimedia Appendix 1*, usage data*). Log data were used to measure the usage rate of the different functions of the portal (eg, messenger), also in relation to demographic data (eg, gender), which should inform the recommendations.

### Questionnaires for Patients

All patients who had provided informed consent to be contacted for evaluation purposes when they gained access to the portal under the terms of use were contacted via clinical staff at the clinics. The data were collected digitally via an online portal and/or using a paper questionnaire. Patients who were no longer in the clinic were contacted by mail. Those who still had access to the portal received an online link. A literature review was conducted, and previous experience related to the planned topics was compiled and coordinated with all project partners (eg, with regard to the planned categorization of questions about the use of the platform in the questionnaire). The questionnaire included self-developed questions on sociodemographic data, usefulness of functions of the portal (free text entry), as well as validated scales regarding satisfaction with use (System Usability Scale, SUS [24,25]), questionnaire on treatment satisfaction (Behandlungszufriedenheit, ZUF-8 [26]), acceptance of online apps (Attitude toward Telemedicine in Psychiatry and Psychotherapy, ATIPP [27]), health literacy (European Health Literacy Questionnaire, HLS-EU-Q16 [28]), and empowerment (patient empowerment [29]). All details can be found in Multimedia Appendix 1, *patients’ perspective: questionnaire*. Data collection took place between October 2024 and January 2025.

### Interviews With Professionals

We invited all trained professionals from the 3 participating clinics via email to take part in an online interview. Between November 2024 and January 2025, professionals were interviewed in guided, semistructured interviews by 1 of 2 trained researchers (LIT and LO). The interviews guides were developed by the project staff in line with previous literature and experiences from similar studies conducted earlier. The interview was pretested internally. The focus of the interviews was on open-ended questions regarding the barriers and facilitators to implementing a patient portal in a mental health hospital setting (Multimedia Appendix 1).

### Development of Recommendations

Based on the quantitative and qualitative study results mentioned above, the core project team (IR, LO, LIT, LK, EG-M) developed a first draft of recommendations for implementing a digital patient portal.

### Expert Consensus Building on Recommendation

This selected group participating in the Delphi process was defined in advance through a participatory process involving the project management and collaboration partners. It included representatives from all relevant groups: mental health care professionals (n=3), psychiatric patient representatives (n=2), project managers for the portal (n=5), and psychiatric hospital managers (n=3). The aim was to identify and prioritize measures that were practical and viable for all stakeholders. Each recommendation was rated against 3 selected criteria using a structured Delphi process [30]. For all recommendations, the importance of the recommendation in general was rated. Further criteria were benefits for patients or professionals, implementation effort, feasibility, and relevance in clinical practice (see Table 2 for an overview).

**Table 2.** Overview of the different criteria for expert rating and allocation for each recommendation for action.

Item	Coding range	Number of recommendation									
		RA1^a^	RA2	RA3	RA4	RA5	RA6	RA7	RA8	RA9	RA10
Importance: How would you rate the importance of the recommendation in general?	1 = “very low” to 5 = “very high”	×	×	×	×	×	×	×	×	×	×
Benefits for patients: What are the benefits of this recommendation for patients?	1 = “no benefit” to 5 = “very high”	×		×	×			×			×
Benefits for professionals: What are the benefits of this recommendation for professionals?	1 = “no benefit” to 5 = “very high”	×		×	×			×			
Implementation effort: How do you estimate the implementation effort?	1 = “very high” to 5 = “very low”		×			×	×		×	×	
Feasibility in clinical practice: How feasible is this recommendation for action in clinical practice?	1 = “very difficult” to 5 = “very easy”		×			×	×		×		
Relevance for clinical practice: How relevant is the following recommendation for action for clinical practice?	1 = “not at all” to 5 = “very much”									×	×

a RA: recommendation for action.

The Delphi process comprised 2 survey rounds: an online real-time survey and a written questionnaire. The group results from the first round were presented anonymously to all participants and discussed within the expert group. The first draft of the recommendations was modified after discussion in the first Delphi round. Participants received a revised version of the recommendation for evaluation in the second (written) Delphi round (up to 4 weeks later).

### Ethical Considerations

The responsible ethics committees approved the study protocol: the Medical Association of North Rhine (Ärztekammer Nordrhein; EK2024186 on September 27, 2025) and the Medical Association of Hesse (Landesärztekammer Hessen; 2024‐3890-zvBO on October 16, 2025). All data protection regulations of the German Federal and State Data Protection Acts and the General Data Protection Regulation (Datenschutz-Grundverordnung) were adhered to within the framework of a data protection concept. Positive data protection votes were obtained in advance from the responsible data protection officers. All patients provided informed consent to be contacted for evaluation purposes when they gained access to the portal under the terms of use. Patients received a compensation of €10 (US $11.50) for their participation. The professionals received compensation of €25 (US $29.50) for their participation.

### Statistical Analysis

#### Overview

We used descriptive statistics to analyze the quantitative data (log data, questionnaire data, and data from the Delphi process ratings), calculating percentages or mean values, medians, and SDs of all frequency measures. For the log data analysis, an additional reduced sample was created, including all patients who had last logged in 4 weeks before the data extraction (51/71). The reduced sample was used to evaluate the duration of use and the number of logins. The entire sample was used for all other variables. Group comparisons were calculated for gender and age. The sample was divided into 2 groups relating to age. Tests for normal distribution were performed, followed by group comparisons (2-tailed *t* test for independent samples for normal distribution and Mann-Whitney *U* test for nonnormal distribution). Chi-square tests were used to analyze differences in frequencies. Missing data were not imputed. IBM SPSS Statistics (version 27) was used for data analyses. Two-sided *P* values of <.05 were regarded as statistically significant.

Participants had the option to provide free-text responses in the data collection section for the questionnaire. The data obtained from these open-ended answers in the patients’ questionnaires were analyzed based on Kuckartz [31]. The data were consecutively coded by 2 coders (IR and LIT) using the MAXQDA software (VERBI), according to predefined categories. The addition of other categories was possible using an inductive approach.

The qualitative interviews with professionals were analyzed based on Kuckartz [31]. Deductive categories based on literature and previous experience were integrated into the interview guideline. These were supplemented by inductive categories that emerged during the interviews. Coding was performed in 2 steps: independent coding by 2 coders (LIT and LO) using MAXQDA software, followed by discussion with a third person (IR) if needed in case of disagreement.

For the analysis of the expert consensus, all ratings of recommendations from the 5-point Likert scale were categorized into positive (categories “rather positive” and “very positive”), neutral, and negative (categories “rather negative” and “very negative”) ratings. We calculated the percentage of participants who agreed on each criterion in a positive or negative way. Consensus was assumed if at least 75% of the ratings were positive or negative in 1 category.

#### Power

As this was a descriptive exploratory study and no sample drawing was involved, we did not perform a power calculation.

#### Data Exclusion

All data were checked for plausibility. Entries from single answers in the app or questionnaires that were not plausible or understandable were excluded from the analysis. Outliers were not omitted from the analysis.

## Results

### Usage Data

#### Sample

Eighty-nine patients used the portal within the study time period. The data from 17 out of 89 (19%) patients were no longer available due to data protection reasons following data deletion after inactivity of more than 6 months. The dataset for the final analysis thus included data from 71 patients, of whom 42 (59%) were female and 29 (41%) were male. No participants were of diverse gender. Patients were, on average, 34.8 years old (range 19‐61 y).

#### Functions Used

The analysis revealed a broad variation regarding the assignment of documents by professionals, as well as the usage and frequency of usage of the portal functions (Table 3): 62% (44/71) of all patients received at least 1 questionnaire from a professional (“assigned”). For 32.4% (n=23/71) of the patients, a therapist in the material pool assigned at least 1 document, while 26.8% (19/71) of the patients had documents assigned to them from the welcome package. All patients (n=71, 100%) used the messenger function. Due to a database storage error, usage data for the appointment or schedule function (acceptance of appointment requests) were only available for 56 out of 71 participants. Of these, 50 (89.3%) patients used the appointments or weekly schedule function. Around half of all patients (38/71, 53.5%) created a diary, but only 18.4% (13/71) shared it with their professional. At least one of the previously assigned questionnaires was completed by 40.9% (18/44) of the patient, with 29.5% (13/44) sharing at least 1 questionnaire with their caregivers. Logbooks and general documents were shared by 19.7% (14/71) of the patients each (Table 3). No significant gender differences were found in terms of the functions used. In terms of age differences, younger patients accepted appointment requests significantly more often than older patients (for 18‐ to 30-year-olds: mean 179.3, SD 70.8 vs for 31‐65-year-olds: mean 101.1, SD 101.3; *P*=.01). There were no other age differences regarding the type of functions used.

**Table 3.** Usage of the different functions of the patient portal (log data).

Variable	Total number	Activity by		Number of users, n (%)	Mean (SD)	Min-max
		Patient	Professional			
Messenger function used (yes)	71	×		71 (100)	—^a^	—
Document assigned (min. 1)	71		×	23 (32.4)	4.4 (3.1)	1-11
Welcome package assigned (min. 1)	71		×	19 (26.8)	3.5 (2.3)	1-8
Diary created (min. 1)	71	×		38 (53.5)	0.7 (1.7)	0-8
Diary edited (min. 1 created)	38	×		10 (26.3)	0.3 (0.8)	0-4
Diary deleted (min. 1)	38	×		8 (21.1)	1.1 (0.7)	0-3
Diary shared (min. 1 assigned)	38	×		31 (81.6)	1.4 (0.6)	1-3
Diary accessed (min. 1 shared)	31		×	8 (25.8)	4.3 (8.2)	0-43
Logbook shared	71	×		14 (19.7)	0.4 (0.8)	0-3
Questionnaire assigned (min. 1)	71		×	44 (62.0)	4.5 (5.3)	1-27
Questionnaire edited (min. 1 assigned)	44	×		18 (40.9)	1.6 (3.3)	0-17
Questionnaire shared (min. 1 assigned)	44	×		13 (29.5)	0.4 (0.7)	0-2
Questionnaire accessed (min. 1 shared)	13		×	7 (53.5)	2.6 (4.1)	0-14

a Not applicable.

#### Duration of Use

The reduced sample comprised 51 portal users who had logged in for the last time 4 weeks before the data extraction. On average, users logged in 9.5 (SD 14.9) times (median 4, IQR 2-7 times). The variability in the number of logins per patient, ranging from 1 to 72, indicated a high variance in frequency of use. On average, the portal was used for 47 (SD 59) days (median 27, IQR 2-62 days). The duration of the use of the portal varied greatly, ranging from 0 to 232 days. A statistically significant positive correlation was found between the total number of logins and the duration of use (*r*=0.758; *P*<.001; Spearman ρ), indicating that users who logged in more frequently were generally active for a longer period of time. Women spent significantly more time on the portal than men did (for women: mean 62.4, SD 65.0 days vs for men: mean 22.9 days, SD 36.9; *P*<.008). Women also logged in significantly more often than men did (number of logins for women: mean 12.9, SD 17.7 vs number of logins for men: mean 4.2, SD 6.4; *P*=.02). There were no significant age differences in the duration of use or the total number of logins within the sample.

### Patient Questionnaire Survey

#### Sample

Twenty-seven patients completed the questionnaire survey (self-reported data), resulting in a response rate of 28%. The majority (17/27, 63%) were female, 9 out of 27 (33%) were male, and 1 identified as diverse. Sixteen out of 27 (59%) participants were under 31 years old, and 11 out of 27 (41%) were aged 31 to 60 years. On average, the patients rated their general IT skills as good (mean 3.4, SD 0.2) and reported having some experience with digital applications in the health sector (mean 3.1, SD 0.2; Multimedia Appendix 1*, Sample characteristics*). Approximately, 51.9% (14/27) of the patients stated that they had been diagnosed with depression or bipolar disorder. Anxiety disorder or a reaction to severe stress was indicated by 29.6% (8/27), while addiction disorder was indicated by 14.8% (4/27). The categories “schizophrenia” and “diagnosis not known” were not selected. “Other diagnosis” was selected by 81.5% (22/27) of the respondents. Approximately, 55.6% (15/27) had used the portal during inpatient hospital treatment, 48.1% (13/27) during day-hospital treatment (day clinic) or home treatment, and 7.4% (2/27) additionally during outpatient treatment (following inpatient or day-hospital treatment). Most (13/27, 48.1%) users had used the portal for less than 1 month, 40.7% (11/27) for 1 to 3 months, and 11.1% (3/27) for more than 3 months.

#### Functions Used and Preferences

On average, patients reported that they had used 2 to 3 functions (mean 2.5, SD 0.2). Approximately 44% (12/27) reported that they had used 2 functions of the portal. The functions mentioned as used most often were the appointments or weekly schedule (21/27, 77.8%), the messenger (14/27, 51.9%), the diary (12/27, 44.4%), the material pool (11/27, 40.7%), and the notes (10/27, 37%). Use of the material pool was rated as very useful or helpful regardless of usage (mean 4.1, SD 1.2). On average, patients rated the portal benefits to date as rather low (mean 2.2, SD 0.2), and their general satisfaction with the portal was 52.7 out of 100 (SD 5.8; median 55, IQR 20-80). Satisfaction with portal usage (SUS) [22,23] was slightly above the scale mean (mean 3.3, SD 0.2). Similarly, treatment satisfaction (ZUF-8) [24] was rated slightly above the scale mean of 3 (mean 3.6, SD 0.1), indicating that the sample was, in general, rather satisfied with their treatment. Regarding patients’ health literacy (HLS-EU-Q16) [26], the mean value was below the midpoint of the scale of 3 (mean 2.2, SD 0.1), giving hints for a rather low health literacy within the sample. For empowerment (patient empowerment) [27], the sample’s value was above the scale mean of 3 (mean 4.3, SD 0.1), with higher values indicating patient sovereignty for patient choice and empowerment. Regarding the acceptance of online apps (ATIPP) [25], the mean value was above the scale mean of 3 (mean 3.76, SD 0.12), indicating that, on average, the sample was more likely to accept online apps. No gender differences were found for the scales reported. However, younger patients (n=16, <31 y) showed significantly higher empowerment values than older patients (n=11, ≥31 y; *U*=38.50, *Z*=−2.448; *P*=.01).

#### Advantages of the Portal (44 Aspects Mentioned by 27 Different Persons) and Barriers to Implementation (44 Aspects Mentioned by 27 Different Persons)

Patients mentioned the following advantages of using an online portal in the open answer section at the end of the questionnaire: optimized communication (13 aspects by 13 persons; Curamenta used as a communication channel and shortened waiting times for responses from professionals) and availability of information (13 aspects by 12 persons; Curamenta provides access to information and materials on diseases, psychoeducation, and relevant content). Patients also mentioned their own activation or motivation (7 aspects by 6 persons) due to the low-threshold offer of materials or information and the structure provided. They stated barriers concerning the usability and functions of the portal (12 aspects by 7 persons), such as an inconvenient app design and complicated login and registration processes. Technical barriers included access issues (10 aspects by 8 persons; the app was unavailable or not working) and internet access issues (no stable Wi-Fi was available on the unit). Furthermore, obstacles on the part of caregivers (5 aspects by 5 persons; eg, the messenger must be kept in view by the contact persons) and patients (3 aspects by 3 persons; eg, preference for writing on paper) were mentioned. Doubts about the secure handling of data and information (4 aspects by 4 persons; data protection and information on the use of data entered into the portal) were also mentioned as obstacles. Suggestions for adjustments or modifications included improving the usability and functionality (eg, enabling the simple tracking of tension and feelings, providing quick access to forms and ensuring the stable functioning of features). Respondents also expressed a desire for more questionnaires for different illnesses and access to their own medical documents (see Multimedia Appendix 1*, Patients’ perspective: Coding system and number of codes* for an overview of all the codes as well as the number of persons who mentioned aspects related to the different codes).

### Professionals’ Perspective

#### Sample and Coding

Fifteen interviews were conducted including 10 with professionals who used the portal and 5 with professionals who did not, although they had been trained to use it. There was no selection since there were exactly 15 responses after the invitation email. No one refused or dropped out during the interview. The interviews were conducted via video call (n=11) or telephone (n=4), with an average duration of 38 (SD 38; range 21‐47) minutes.

The interviewees included 4 psychiatrists, 4 psychologists, 6 nurses, and 1 physical therapist. Of the professionals, 8 (53%) were male and 7 (47%) were female. Most of the participants worked in an inpatient setting. The coding tree contained the following overarching categories: “general experiences of nonusers and users,” “reasons for nonusage,” “description of usage in clinical care,” “user-friendliness for patients,” “experiences of professionals,” “advantages or disadvantages of usage,” “acceptance,” “facilitators or barriers for implementation,” “requirements for implementation in routine clinical care,” and “desired adjustments to the platform” (for details, see Multimedia Appendix 1). According to the theoretical approach [31], topics were identified in advance, but new topics could also arise from the data. The coding tree included 690 quotations. Theoretical saturation was achieved during the analysis. The interviews mainly focused on the professionals’ perceptions of barriers and facilitators.

A total of 135 quotations related to the implementation process facilitators were mentioned throughout the interviews. These were categorized as either patient-related (n=34; mentioned by 13 out of 15 participants), professional-related (n=20; mentioned by 13 participants), organizational (n=29; mentioned by 15 participants), structural (n=19; mentioned by 10 participants), or technical (n=27; mentioned by 11 participants).

The barriers to implementing or using the platform portal (157 in total) were divided into the following categories:

- Patient-related (34 aspects, mentioned by all 15 participants)
- Professional-related (30 aspects, mentioned by 12 participants)
- Structural (27 aspects, mentioned by 12 participants)
- Organizational (21 aspects, mentioned by 13 participants)
- Technical (29 aspects, mentioned by 12 participants)
- Others (6 aspects, mentioned by 5 participants; Multimedia Appendix 1*, Coding system from qualitative interviews with professionals*).

#### Facilitators and Barriers of Implementation

Patient-related aspects that were found to have a beneficial effect on the use of the portal were basic digital affinity or experience in using digital apps; good language skills, due to the text-based structure of the app; younger age; and a higher level of functionality. This is illustrated in interview 8:

*Well, a younger age […] is a certain pull factor, so to speak. And there is perhaps more interest. And of course a certain affinity for technology on the patient side. Of course, the general motivation for therapy, right? In other words, how much do I want to engage with the treatment outside of the therapy services? So the higher the motivation for therapy and change, the higher, I think, the willingness to use Curamenta as an additional service.*

*[translated from German]*

Patients in a stabilized condition and with an increased willingness to change were also more likely to actively use the portal. Recognizable therapeutic added value for patients and stable, trusting therapeutic relationships were cited as key prerequisites for use. It was reported that day clinic settings, which involve more independent patients and longer stays, tended to encourage active use of the portal. With regard to patient-related barriers, the relevant hindering factors mentioned were the type of disorder (eg, severe psychosis, media addiction, or an acute event) and individual patient characteristics (eg, concentration problems, difficulty structuring oneself or a lack of previous IT experience). Other reported barriers included an advanced age of patients, a short length of stay, and patients’ general rejection of the portal.

On the part of the professionals, their own digital affinity and openness to new things were emphasized, as well as availability in terms of time (for use in everyday clinical practice, as well as for familiarization and training). When the use of the portal was perceived as beneficial, this increased engagement, as the portal was seen as making work easier. It was emphasized that it would be beneficial for the materials to be tailored more closely to professionals’ needs, for example, at the beginning of treatment. With regard to barriers relating to professionals, the following were mentioned: a lack of affinity with technology, low motivation due to a lack of relevant or suitable content on the portal, and concerns about unclear responsibilities in the event of the illness of a therapist. The additional time burden, high workload, and a lack of perceived added value of the portal for professionals’ own work represented a barrier to implementation. An illustration of a professional-related barrier from interview 4 was the high workload of professionals:

*Yes, it’s always an extra. So it’s not as if you “save time” somewhere else. It’s quite clear that it has to be packed into the daily work routine somehow, where it perhaps doesn't fit in at all.*

[translated from German]

At an organizational level, professionals stated that having a shared understanding of the objectives and use of the portal within the team was helpful. Integrating the portal into existing routines, such as morning meetings, patient rounds, and team meetings, was considered particularly effective. Within the team organization, personnel bottlenecks and a lack of time and resources (eg, for inductions and additional meetings) were mentioned as barriers. In some cases, the fact that not all team members had received training was a barrier, exacerbated by staff turnover. Unclear allocation of responsibilities within the team, the lack of shared use of logins by medical and psychological staff, and negative experiences with nonfunctioning technology had a demotivating effect on the teams.

*Well, I think that’s the biggest organizational obstacle that you have to somehow fit it into your daily routine*.

[Interview 15 (translated from German)]

With regard to hospital structure, it was emphasized that support from superiors was crucial. It was emphasized that all individuals responsible for the portal should actively support it and demonstrate their commitment to its implementation. Support from central staff units was also positively emphasized. Clear timetables and defined processes for introducing the portal to various clinical areas were also considered helpful. It was suggested that an increased presence of the portal in cross-clinic exchanges (eg, meetings of assistant doctors or therapeutic specialist teams) would be helpful. The failure to integrate the portal into existing processes was often cited as a barrier. It was reported that the portal was not fully introduced in the clinics, meaning that some patients had access while others did not, which was perceived as unfair. The scarcity of personnel and limited time resources in everyday clinical practice were also described as hurdles, for example, in cases of staff rotation, personnel bottlenecks, or high workloads.

*That means making it easier if the medical director communicates very clearly, especially on the treatment side, […] And there will be very clear framework conditions/that is, a tighter corset on how it is to be used for which patient. […] for example, will no longer be given in paper form, if patients want it, they have to register, then they get it digitally. So, if there’s a bit more stringency in there.*

[Interview 11 (translated from German)]

The technical aspects considered central to successful implementation were a sufficient and free supply of Wi-Fi and suitable end devices for patients and professionals. In addition, there was a desire for a professional app and improved interfaces with the hospital information system. Beneficial further developments mentioned included automated feedback and push notifications from the portal for patients and professionals, as well as on-site technical support within the clinic.

Issues include a lack of internet access on the hospital premises, missing or inadequate end devices for patients, difficulties logging in, a lack of app compatibility for certain modules, and repeated interface failures with hospital information systems.

*Other aspects would be, for example, the problem of, yes, the interface between the HIS and Curamenta, which is repeatedly disrupted and therefore also greatly reduces the previously mentioned added value. But also, precisely, the aspects that we have already mentioned, for example extensive data protection, extensive approvals, naturally provide a strong obstacle in the general process.*

Interview 1 [translated from German]

### Recommendation Development and Expert Consensus Building on Recommendations

Based on the log data, the questionnaire data, and the qualitative study results mentioned above, the project team (IR, LO, LIT, LK, EG-M) developed recommendations for implementing a digital patient portal (Table 4). Therefore, all results from the different work packages were discussed intensively within the core project team, and a first set of 24 recommendations was drafted in an inductive way. This initial set was discussed and condensed to the 10 most obvious and central recommendations, which were considered most important by the majority of the team members. These were grouped into 5 overarching topics for action: Implementation (4), technical or structural aspects (2), usability (1), communication or training (1), and evaluation (2). Recommendations were presented and discussed in a common digital meeting with the expert group selected for the expert consensus process.

**Table 4.** Overview of recommendations for implementation.

Thematic field and number	Informed by	Recommendation for action	% of consensus by expert group^a^
Implementation			
1	Quan^b^, Qual^c^	The online portal should already be technically stable and largely error-free in use before it is implemented on a large scale in routine clinical care.	88.9
2	Quan, Qual	The online portal should only be implemented after it has been successfully tested in pilot projects. Particular emphasis should be placed on the structural/staffing requirements that must be provided by those responsible/clinic management before implementation and on an ongoing basis.	88.9
3	Quan, Qual	A largely functioning app function of the online portal should be available at the start of implementation.	88.9
4	Quan, Qual	There should be sufficient added value in terms of content for patients and professionals (eg, diagnostic materials, psychoeducational content, working materials for therapies, relaxation exercises, standardized progress monitoring) before an online portal (which should serve not only organizational/administrative but also content-related purposes) is implemented on a large scale in routine clinical care.	88.9
Technical-structural aspects			
5	Qual	It is recommended that all relevant persons be given low-threshold (technical) access to the online portal in the clinics. Particular emphasis should be placed on (1) the availability of free Wi-Fi on participating wards or in participating clinics and (2) the provision of mobile, digital devices for practitioners on the hospital units that can be used in patient consultations.	88.9
5a^d^	Qual	It is recommended that all relevant persons be given low-threshold (technical) access to the online portal in the clinics. Particular emphasis should be placed on the availability of free WIFI on participating wards or in participating clinics.	88.9
5b^d^	Qual	It is recommended that all relevant persons be given low-threshold (technical) access to the online portal in the clinics. The provision of a shared pool of mobile, digital end devices for multi-professional teams on the hospital units, which can be used in patient consultations, should be particularly emphasized.	88.9
6	Log^e^, Qual	It is recommended that the online portal is linked to the hospital information system (HIS)^f^ in such a way that jump/log-in processes are simplified and desired content (eg, diagnostics, progress measurements) can be transferred from the online portal to the HIS and thus documented.	77.8
Usability/user friendliness			
7	Log, Quan, Qual	The portal should be designed and operated as simply and intuitively as possible. Particularly noteworthy are (1) the user-friendly implementation of the online portal, even for less IT-savvy people, (2) the integration of functions known to the general public (eg, push messages, search or filter function in the material pool), and (3) attractive design for patients (eg, gamification).	88.9
Communication/ Training			
8	Log, Qual	Treatment providers should be informed at an early stage and in an appropriate format by hospital managers and trained at regular intervals by expert staff (eg, permanent staff units) in order to avoid uncertainty on the part of treatment providers and increase motivation.	100
Evaluation			
9	Log	It is recommended that online portals be designed from the outset in such a way that anonymized usage data can be easily and regularly evaluated and used to monitor the portal without significant additional effort or costs. When evaluating data, the usage data of practitioners should also be recorded and analyzed anonymously.	55.5
10	Log, Quan	It is recommended that patient satisfaction (eg, at the time of discharge) be routinely recorded and analyzed anonymously.	88.9

a Rating of importance of all recommendations by expert group (% of rating 4 [high importance] and 5 [very high importance]), second consensus round, n=9.

b Quan: questionnaire data.

c Qual: interview data.

d New recommendations from the second round.

e log: log data.

f HIS: hospital information system.

Thirteen people participated in the first Delphi round, one of whom ended the evaluation prematurely. Nine people participated in the second Delphi round. These recommendations were rated according to 3 previously selected criteria (Table 2). Following a discussion after the first Delphi round, the fifth recommendation was divided into 2 parts to simplify the assessment process. No other changes were made. All recommendations were highly rated in terms of importance. Recommendation 1 (“The online portal should be technically stable and largely free of errors before it is implemented on a large scale in routine clinical care”) received the highest importance rating (mean 4.89, SD 0.33). Consensus among experts was high throughout most recommendations, especially regarding the ratings of importance of the recommendation (range 77.8%-100% consensus of the experts for 9 out of the 10 recommendations) and the benefits for patients (77.8%‐100% consensus). Other criteria were rated more heterogeneously (see Table 4, and for more details, see Multimedia Appendix 1*, Expert consensus*).

## Discussion

### Main Results

The overarching goal of the study was to identify the barriers to and facilitators of implementing an online patient portal in a mental health hospital setting in Germany. Curamenta is an example of a digital gateway portal, which provides a range of communication and survey functions for psychiatric patients and professionals. The portal was still in the early stages of implementation when the study was performed. Using a mixed methods approach, we identified technical, structural, as well as patient- and professional-related facilitators and barriers. Using methods of structured consensus building, we identified a set of 10 recommendations, which may guide future similar implementation projects in other mental health care settings.

The results from the various work packages demonstrate that implementing an online portal in a mental health hospital setting in Germany was feasible. The different data sources offered information about platform usage and preferences and the interplay between the different sources resulted in the integration of study results and the development of 10 recommendations for implementation.

Log data from 71 patients indicated individually differential use of the portal’s various functions. This is consistent with literature reporting variation in usage data across different user groups (eg, [6]). However, it is unclear whether higher usage correlates with a greater impact on health outcomes [32]. Since we did not measure health outcomes, we cannot comment on this issue. The strength of our study was to identify characteristic patterns of usage of the online patient portal, which may inform future implementation projects about desirable processes and structures for digital gateway portals in mental health care. Future (larger, longitudinal) studies should attempt to provide more detailed information about specific usage and the needs of different patient groups and settings.

Active users tended to be younger and predominantly female. Gender-specific analyses in this area are rare [33], but evidence from other health care disciplines suggests that women may experience a higher sense of control when using a patient portal to assist with care planning [34]. Similar studies involving male or diverse patients are not available but would be necessary to establish.

Younger, female, technophile users mostly completed the patient survey. They showed mixed satisfaction with the portal. The use rates of the features varied, which is consistent with other studies (eg, [12]). The contributing factors to this variation (eg, diagnosis) remain unclear, as subgroup analysis was not possible due to the small sample size in this study. Etingen et al [35] discovered in a retrospective analysis that having anxiety disorders, posttraumatic stress disorder, and depression was associated with a greater likelihood of portal use. A scoping review by Rabbani et al [36] evaluated characteristics of portal use specifically for individuals with depression, including the provision of tailored services and a messaging feature between patients and providers. This aligns with a review by Durocher et al [33], which indicated that health equity factors, such as income, lack of access to private insurance, and health literacy, were mentioned as potential barriers to accessing and using patient portals. We also obtained preliminary evidence, suggesting that tailoring the use of digital interventions (eg, providing more differentiated material) according to patient needs may be beneficial. Furthermore, we collected patient responses regarding the advantages of using a patient portal and the barriers to its implementation. Patients reported advantages in terms of communication and access to relevant information.

The barriers mentioned by the professionals (eg, overwhelming workloads, insufficient time allocated for working with digital tools, inadequate time adjustments, and a lack of ongoing training) were comparable to those reported in other studies on the implementation of e-mental health elements in inpatient settings (eg, [37]). Perceived usefulness has been shown to be crucial for implementation success in previous studies [6]. Barriers related to the inpatient setting, such as the severity of the disease or a lack of motivation, have also been identified in previous studies [38] and may be particularly important for the implementation of digital tools in this setting. Professionals reported on the barriers and promoting factors that should be considered when setting priorities for the portal’s further development and implementation. Professionals’ adoption of the portal may impact patients’ adoption [6]. Previous studies from Germany reported that clinicians have a low level of experience, computer skills, and knowledge of e-mental health interventions [12,39]. Additionally, characteristics such as providing compassionate care and fostering interprofessional collaboration may enhance treatment experiences [7,40]. It has become clear that support for patients and professionals is needed to achieve the portal’s full potential and encourage engagement.

The combination of different data sources led to more insights into usage and preferences and mixing methods helped to combine perspectives from different users. By integration of study results, 10 recommendations for implementation could be developed. All 10 recommendations for implementing online portals in a mental health hospital setting were rated as important (Table 4). Generally, ratings related to patient needs were higher than those related to professional needs. Furthermore, the ratings of realization and expenditure indicated the relatively high estimated effort required for the implementation of a patient portal. Overall, the results of the study may help to advance the implementation of patient portals, particularly in the field of mental health care.

### Limitations

We conducted a naturalistic study during the initial implementation phase of a digital patient portal in psychiatric hospital setting. The initial group of patients may not be representative of the inpatient population in psychiatric hospitals, as they may have been very motivated individuals. The evaluation process was adapted to the ongoing implementation and was not designed in advance, which resulted in limited data availability for evaluation [6]. Awareness of barriers to patient portal use, such as severe health issues and health equity factors, is an important consideration for the use and uptake of patient portals [33]. Our sample sizes were rather small, which was related to the early implementation phase and the selection of 3 hospitals being in the starting phase of data collection for logistical reasons. The availability of log data was limited since the database was not originally built for detailed evaluation during the launch of the portal. Usage intensity and duration of use may have been influenced by the degree to which professionals motivated patients or interacted with them on the platform, but we were not able to assess this within the study context. As the usage of the portal increases, the next step in its implementation would be to enrich log data with more variables (eg, diagnosis). For our small study with a limited number of patients, we had to keep different data sources separate to avoid deanonymization. It was not possible to collect longitudinal data due to the limited timeframe of the study. However, it seems helpful to continuously monitor the implementation process and clarify open research questions regarding the possible effects of portal usage on clinical outcomes and patient satisfaction. For the qualitative analysis, no interrater reliability was calculated. Finally, our study lacks a control group without access to the patient portal.

### Comparison With Prior Work

To our knowledge, this is the first study to combine quantitative and qualitative data collection from the implementation of an online portal in Germany and to develop evidence- and consensus-based recommendations to advance the implementation of a digital gateway portal for mental health care in a mental health hospital setting in Germany. We confirm previous findings that designing digital mental health interventions requires designing for specific populations and addressing specific needs and that this may increase acceptance and engagement [41]. The results from our study are in line with previous findings from other large health care delivery systems, indicating that patients prescribed digital interventions are predominantly female and young [42]. This suggests gender- and age-related gaps in digital mental health implementation, gaps that professionals trained in and using a nonselective gateway portal like Curamenta may help to overcome. Online portals may be strengthened in this role by integrating specific content or usability support for male patients and older patients. Our study findings are also corroborated by the results of previous stakeholder assessments in Germany regarding potential barriers and facilitators of digital mental health implementation [14,39], in which professional training requirements and lack of professional experience emerged as key challenges. Our work provides useful evidence and experiences for informing the further development of conceptual implementation frameworks such as the inpatient digital mental health implementation framework developed by Westheimer and colleagues [43], who suggested to balance the design of the digital intervention with the development of end user resources, a key finding from our study.

### Conclusions

Besides informing the future process of implementing Curamenta, the recommendations for action generated from this study may be used to help implement other patient portals in the psychiatric hospital settings. Given that the challenges in other countries appear similar to the situation in Germany, the recommendations seem to be highly generalizable.

## Supplementary material

10.2196/82450 Multimedia Appendix 1 Variables and additional results.

10.2196/82450 Checklist 1 GRAMMS checklist.

10.2196/82450 Checklist 2 COREQ checklist.

## Abbreviations

ATIPP: Attitude toward Telemedicine in Psychiatry and Psychotherapy
COREQ: Consolidated Criteria for Reporting Qualitative Research
GDG: Society for digital health [Gesellschaft für Digitale Gesundheit]
GRAMMS: Good Reporting of a Mixed Methods Study
HLS-EU-Q16: European Health Literacy Questionnairehealth literacy questionnaire
ZUF-8: Questionnnaire on treatment satisfaction [Behandlungszufriedenheit]

## References

[1] Lin CT, Wittevrongel L, Moore L, Beaty BL, Ross SE (2005). An internet-based patient-provider communication system: randomized controlled trial. J Med Internet Res.

[2] Chen L, Chuang LM, Chang CH (2013). Evaluating self-management behaviors of diabetic patients in a telehealthcare program: longitudinal study over 18 months. J Med Internet Res.

[3] Andersson G, Carlbring P, Titov N, Lindefors N (2019). Internet interventions for adults with anxiety and mood disorders: a narrative umbrella review of recent meta-analyses. Can J Psychiatry.

[4] Philippe TJ, Sikder N, Jackson A (2022). Digital health interventions for delivery of mental health care: systematic and comprehensive meta-review. JMIR Ment Health.

[5] Diel A, Schröter IC, Frewer AL (2024). A systematic review and meta analysis on digital mental health interventions in inpatient settings. NPJ Digit Med.

[6] Zhang T, Shen N, Booth R, LaChance J, Jackson B, Strudwick G (2022). Supporting the use of patient portals in mental health settings: a scoping review. Inform Health Soc Care.

[7] Shin HD, Durocher K, Lo B (2023). Impact of a mental health patient portal on patients’ views of compassion: a mixed-methods study. BMC Digit Health.

[8] Meier-Diedrich E, Turvey C, Wördemann JM, Speck J, Weibezahl M, Schwarz J (2025). Patient-health care professional communication via a secure web-based portal in severe mental health conditions: qualitative analysis of secure messages. JMIR Form Res.

[9] Irizarry T, DeVito Dabbs A, Curran CR (2015). Patient portals and patient engagement: a state of the science review. J Med Internet Res.

[10] Kooij L, Groen WG, van Harten WH (2018). Barriers and facilitators affecting patient portal implementation from an organizational perspective: qualitative study. J Med Internet Res.

[11] Uribe Guajardo MG, Baillie A, Louie E (2022). The evaluation of the role of technology in the pathways to comorbidity care implementation project to improve management of comorbid substance use and mental disorders. J Multimorb Comorb.

[12] Bass E, Garabrant J, Salyers MP, Patterson S, Iwamasa GY, McGuire AB (2023). eHealth use on acute inpatient mental health units: implementation processes, common practices, and barriers to use. Adm Policy Ment Health.

[13] Drozd F, Vaskinn L, Bergsund HB, Haga SM, Slinning K, Bjørkli CA (2016). The implementation of internet interventions for depression: a scoping review. J Med Internet Res.

[14] Sander J, Bolinski F, Diekmann S (2022). Online therapy: an added value for inpatient routine care? Perspectives from mental health care professionals. Eur Arch Psychiatry Clin Neurosci.

[15] Richter LE, Machleit-Ebner A, Scherbaum N, Bonnet U (2023). How effective is a web-based mental health intervention (Deprexis) in the treatment of moderate and major depressive disorders when started during routine psychiatric inpatient treatment as an adjunct therapy? A pragmatic parallel-group randomized controlled trial. Fortschr Neurol Psychiatr.

[16] Dorow M, Stein J, Förster F (2018). Der komplementäre Einsatz des internetbasierten Selbstmanagementprogramms „moodgym“ bei Menschen mit depressiven Erkrankungen in der stationären Versorgung – die Perspektive von Patienten und Behandlern [Article in German]. Psychiat Prax.

[17] Proctor EK, Landsverk J, Aarons G, Chambers D, Glisson C, Mittman B (2009). Implementation research in mental health services: an emerging science with conceptual, methodological, and training challenges. Adm Policy Ment Health.

[18] Zielasek J, Reinhardt I, Schmidt L, Gouzoulis-Mayfrank E (2022). Adapting and implementing apps for mental healthcare. Curr Psychiatry Rep.

[19] Gaebel W, Lukies R, Kerst A (2021). Upscaling e-mental health in Europe: a six-country qualitative analysis and policy recommendations from the eMEN project. Eur Arch Psychiatry Clin Neurosci.

[20] Waltz TJ, Powell BJ, Fernández ME, Abadie B, Damschroder LJ (2019). Choosing implementation strategies to address contextual barriers: diversity in recommendations and future directions. Implement Sci.

[21] Reinhardt I, Holsten R, Zielasek J, Kuhlmann L, Gouzoulis-Mayfrank E (2024). Implementation of an electronic patient portal in routine mental health care of hospitals in Germany—evaluation of attitudes of healthcare providers. BMC Health Serv Res.

[22] O’Cathain A, Murphy E, Nicholl J (2008). The quality of mixed methods studies in health services research. J Health Serv Res Policy.

[23] Tong A, Sainsbury P, Craig J (2007). Consolidated criteria for reporting qualitative research (COREQ): a 32-item checklist for interviews and focus groups. Int J Qual Health Care.

[24] Brooke J, Jordan PW, Thomas B, McClelland IL, Weerdmeester B (1996). Usability Evaluation in Industry.

[25] Rummel B System usability scale – jetzt auch auf deutsch. SAP Community.

[26] Schmidt J, Lamprecht F, Wittmann WW (1989). Zufriedenheit mit der stationären Versorgung. Entwicklung eines Fragebogens und erste Validitätsuntersuchungen [Article in German]. PPmP: Psychotherapie Psychosomatik Medizinische Psychologie.

[27] Tonn P, Reuter SC, Kuchler I (2017). Development of a questionnaire to measure the attitudes of laypeople, physicians, and psychotherapists toward telemedicine in mental health. JMIR Ment Health.

[28] Jordan S, Hoebel J (2015). Health literacy of adults in Germany: findings from the German Health Update (GEDA) study [Article in German]. Bundesgesundheitsblatt Gesundheitsforschung Gesundheitsschutz.

[29] Kerber A, Beintner I, Burchert S, Knaevelsrud C (2023). Effects of a self-guided transdiagnostic smartphone app on patient empowerment and mental health: randomized controlled trial. JMIR Ment Health.

[30] Niederberger M, Deckert S (2022). The Delphi technique: methodology, variants and usage examples [Article in German]. Z Evid Fortbild Qual Gesundhwes.

[31] Kuckartz U (2018). Qualitative Inhaltsanalyse Methoden, Praxis, Computerunterstützung.

[32] Lo B, Shin HD, Kemp J (2024). Shifting mindsets: the impact of a patient portal on functioning and recovery in a mental health setting. Can J Psychiatry.

[33] Durocher K, Shin HD, Jackson KT, Strudwick G (2024). Women’s experiences of using patient portals in healthcare settings: a rapid review. BMC Womens Health.

[34] Mohsen K, Kildea J, Lambert SD, Laizner AM (2021). Exploring cancer patients’ perceptions of accessing and experience with using the educational material in the opal patient portal. Support Care Cancer.

[35] Etingen B, Hogan TP, Martinez RN (2019). How do patients with mental health diagnoses use online patient portals? An observational analysis from the Veterans Health Administration. Adm Policy Ment Health.

[36] Rabbani M, Nasiri M, Mowla A, Sharifian R (2023). Mental health patient portals aimed at depression: a picture close to reality. Stud Health Technol Inform.

[37] Gupta N, Leuba S, Seifritz E, Berger T, Kawohl W (2024). Resources, support, and integration as potential barriers and facilitators to the implementation of blended therapy in the routine care of inpatients: a qualitative study. Front Psychiatry.

[38] Van Assche E, Bonroy B, Mertens M (2022). E-mental health implementation in inpatient care: exploring its potential and future challenges. Front Digit Health.

[39] Weitzel EC, Schwenke M, Schomerus G (2023). E-mental health in Germany—what is the current use and what are experiences of different types of health care providers for patients with mental illnesses?. Arch Public Health.

[40] Durocher K, Shin HD, Lo B, Chen S, Ma C, Strudwick G (2023). Understanding the role of patient portals in fostering interprofessional collaboration within mental health care settings: mixed methods study. JMIR Hum Factors.

[41] Borghouts J, Pretorius C, Ayobi A, Abdullah S, Eikey EV (2023). Editorial: factors influencing user engagement with digital mental health interventions. Front Digit Health.

[42] Ridout SJ, Ridout KK, Lin TY, Campbell CI (2024). Clinical use of mental health digital therapeutics in a large health care delivery system: retrospective patient cohort study and provider survey. JMIR Ment Health.

[43] Westheimer JL, Moukaddam N, Lindsay JA (2023). Technology implementation for mental health end users: a model to guide digital transformation for inpatient mental health professionals. JMIR Ment Health.

[44] DeepL.

