# Supplementary material for: Barriers and Facilitators for the Implementation of an Online Portal in Hospital Mental Health Care: Implementation Study
Source: JMIR Form Res. 2026 May 19;10:e82450. doi: 10.2196/82450 (PMC13186438; doi:10.2196/82450)
Supplement: Multimedia Appendix 1 [file formative-v10-e82450-s001.docx]

**Online Supplement: Evaluation of barriers and facilitators for the implementation of an online portal for patients in psychiatric hospitals**

1. **Usage data: List of variables (on patient level)**

| Description of variables |
| --- |
| Gender |
| Year of birth |
| Hospital location |
| Patient ID |
| Date of the last hospital discharge |
| Date and time of the last login |
| Total number of logins |
| Date and time of the first login |
| Did the patient use the messenger function? |
| How often has the patient worked on diaries? |
| How many diaries has the patient created? |
| How many diaries did the patient delete? |
| Number of documents assigned by the practitioner |
| Number of welcome packages assigned by the practitioners |
| Number of questionnaires assigned by the practitioner |
| How often has the patient completed a questionnaire? |
| How many appointment requests has the patient made? |
| How often did the patient share questionnaires? |
| How often did the patient share documents? |
| How often did the patient share their logbook? |
| How often did the patient share their diary? |
| Number of times practitioners have accessed shared questionnaires |
| Number of times practitioners accessed shared diaries |

1. **Patients’ perspective: Questionnaire** [incl. answer categories]

General information: The following are general questions about you, your knowledge of digital applications and your experience with the Curamenta online platform.

1. How old are you? [*younger than 31 years; 31-60 years old; older than 60 years]*
2. Which gender do you consider yourself to be? [*female; male; diverse]*
3. How would you rate your general IT knowledge and skills? [*very bad, bad, average, good, very good]*
4. How much experience do you already have with digital applications in the health sector (e.g., fitness trackers, apps)? [*very little experience, little experience, some experience, much experience, very much experience]*
5. In which setting did you use Curamenta or are you currently using Curamenta*? [Inpatient setting, day clinic, outpatient setting]*
6. For which mental illness(es) are/were you undergoing treatment while using Curamenta? (Multiple answers are possible) [*Depression/bipolar disorder, schizophrenia, addictive disorder, anxiety disorder or PTSD, other, unknown]*
7. How long have you (actively) used Curamenta? [*< 1 month, 1-3 months, >3 months]*
8. Which areas or functions of Curamenta have you used/are you using? [*Diary, appointments/weekly schedule, messenger, notes, material pool]*
9. How helpful/useful do you find Curamenta’s areas or functions? [*Not at all, rather not, neither, somewhat, very much]*
10. What other Curamenta functions would you like to see? [*Open question]*
11. Have you used Curamenta before using the patient-therapist level? (e.g. forums, self-tests)? [*Yes, no, don’t know]*
12. How would you rate the overall benefit of Curamenta for yourself to date? [*very low, low, rather high, very high]*

The following questions deal with the advantages and obstacles of using online platforms, such as Curamenta, as well as the requirements and use of online platforms.

1. What advantages do you see for yourself as a patient through the use of online platforms, such as Curamenta, in the hospital environment? [*Open question]*
2. What obstacles do you see to the use of online platforms, such as Curamenta, in the hospital environment? [*Open question]*
3. What do you consider to be important prerequisites for the use of online platforms, such as Curamenta, in the hospital environment? [*Open question]*
4. What are the fundamental arguments against the use of online platforms, such as Curamenta, in the hospital environment? [*Open question]*

Questionnaire to measure patient satisfaction *(ZUF-8 [33]): 8 items;* Satisfaction with the use of the portal: *System Usability Scale (SUS; [34]): 10 items*

Please tick the answer that most closely matches: How satisfied are you in general with the use of Curamenta? (0 = very dissatisfied, 100 = very satisfied)

*Scale ‘European health literacy questionnaire’ (HLS-EU-Q16 [35]): 16 items; Scale ‘Patient Empowerment’ (PS; [36]): 10 items; Scale ‘Attitude toward Telemedicine in Psychiatry and Psychotherapy’ (ATIPP; [37]): 8 items*

*Do you have any comments on Curamenta or the implementation of online applications that you would like to share with us?*

**Thank you for your participation!**

1. **Sample characteristics (questionnaire filled out by patients)**

| Demographic and usage data | N | % of the sample | M | SD | med | range |
| --- | --- | --- | --- | --- | --- | --- |
| Gender  Female  Male  Divers | 17  9  1 | 63.0  33.3  3.7 |  |  |  |  |
| Age group  > 31 years  31 to 60 years  < 60 years | 16  11  0 | 59.3  40.7  - |  |  |  |  |
| IT skills  *(scale: 1 very poor to 5 very good)* | 27 | 100 | 3.4 | 0.2 | 4 | 2-5 |
| How much experience do you have with digital health applications (e.g. fitness tracker, app)?  *(scale: 1 very little to 5 very much)* | 27 | 100 | 3.1 | 0.2 | 3 | 1-5 |
| Diagnosis (multiple answers possible)  Depression or bipolar disorder  Anxiety disorder or PTBS  Psychoactive substance use  Schizophrenia  Diagnosis not known  Other | 48  14  8  4  0  0  22 | 51.9  29.6  14.8  -  -  81.5 |  |  |  |  |
| Setting during Curamenta use (multiple answers possible)  Inpatient treatment  Day clinic/home treatment  Outpatient treatment | 30  15  13  2 | 55.6  48.1  7.4 |  |  |  |  |
| Duration of use of the platform (number of patients)  < 1 month  1-3 months  > 3 months | 13  11  3 | 48.1  40.7  11.1 |  |  |  |  |
| Rating of benefit of use  *(scale range: 1 very low to 4 very high)* | 27 |  | 2.2 | 0.2 | 2 | 1-4 |
| General satisfaction of usage  *(scale range: 0 very unsatisfied to 100 very satisfied)* | 26 |  | 53 | 5.8 | 55 | 10-100 |
| Number of functions used per patients | 27 |  | 2.5 | 0.2 | 2 | 1-5 |
| Functions used (multiple answers possible)  Appointments/weekly schedule  Messenger  Diary  Material pool  Notes | 21  14  12  11  10 | 77.8  51.9  44.4  40.7  37.0 |  |  |  |  |
| Rating of usefulness of functions – only users of the function  *(scale: 1 not useful at all to 5 very useful*)  Appointments/weekly schedule  Messenger  Diary  Material pool  Notes | 25  24  27  25  24 |  | 3.95  4.43  4.33  4.45  3.89 | 1.43  1.02  0.89  0.93  0.78 |  |  |
| Rating of usefulness of functions independently of actual usage  *(scale: 1 not useful at all to 5 very useful)*  Appointments/weekly schedule  Messenger  Diary  Material pool  Notes | 11  14  21  12  9 |  | 3.93  3.92  3.60  4.12  3.46 | 1.30  1.35  1.16  1.20  1.18 |  |  |
| SUS | 27 |  | 3.33 | 0.16 | 3.1 | 1.9-4.7 |
| ZUF-8 | 27 |  | 3.56 | 0.08 | 3.5 | 2.8-4.0 |
| ATiPP | 27 |  | 3.76 | 0.12 | 3.9 | 1.9-4.8 |
| HLS-EU-Q16 | 27 |  | 2.22 | 0.09 | 2.2 | 1.4-3.0 |
| PS | 27 |  | 4.30 | 0.11 | 4.3 | 2.6-5.4 |

M = mean value; n = number of participants; % = percentage; SD = standard deviation; N = total number; IT = information technology; SUS = satisfaction with use; ZUF-8= treatment satisfaction; ATiPP = acceptance of online applications; HLS-EU-Q16 = health literacy; PS = empowerment; StäB = home treatment

1. **Patients’ perspective: Coding system and number of codes**

| Coded items | | | 227 (No. of persons out of 27) |
| --- | --- | --- | --- |
|  | **Desired customization of Curamenta** | | 43 (27) |
|  |  | Usability and functionality | 20 (11) |
|  |  | Content | 8 (5) |
|  |  | other | 3 (3) |
|  |  | none | 2 (2) |
|  |  | no answer | 10 (10) |
|  | **Advantages of using Curamenta** | | 44 (26) |
|  |  | Optimized communication | 13 (13) |
|  |  | Digitalization | 3 (3) |
|  |  | (Digital) design | 6 (6) |
|  |  | Availability of information | 13 (12) |
|  |  | Patient activation | 7 (6) |
|  |  | no answer | 2 (2) |
|  | **Obstacles to using Curamenta** | | 44 (27) |
|  |  | Barriers on the professionals’ side | 5 (5) |
|  |  | Technical access | 10 (8) |
|  |  | Secure handling of data and education | 4 (4) |
|  |  | Usability and functions | 12 (7) |
|  |  | Internet access | 6 (6) |
|  |  | Patient characteristics | 3 (3) |
|  |  | none | 1 (1) |
|  |  | no answer (obstacles/against use) | 3 (3) |
|  | **Prerequisite for using Curamenta** | | 44 (27) |
|  |  | Continuous improvement in technical implementation | 6 (6) |
|  |  | Secure and informed handling (of data) | 11 (10) |
|  |  | Characteristics (e.g. IT knowledge/ competence) | 12 (10) |
|  |  | Contact person | 2 (2) |
|  |  | Handling/design | 5 (4) |
|  |  | Internet access | 4 (4) |
|  |  | none | 1 (1) |
|  |  | no answer/unclear wording | 3 (3) |
|  | **Reasons against use** | | 27 (27) |
|  |  | Obstacles on the professionals’ side | 6 (6) |
|  |  | Technical access | 1 (1) |
|  |  | Secure handling of data and information | 4 (4) |
|  |  | Usability and functions | 2 (2) |
|  |  | Technical infrastructure | 2 (2) |
|  |  | Patient characteristics | 3 (3) |
|  |  | none | 7 (7) |
|  |  | no answer | 4 (4) |
|  | **Other notes** | | 7 (7) |
|  |  | none | 1 (1) |
|  |  | no answer | 15 (15) |

No = Number

**5. Coding system from qualitative interviews with professionals**

| Category | Subcategory | Codings  (No. of persons out of 15) | Content | Example |
| --- | --- | --- | --- | --- |
| Facilitators for implementation* | | 135 (15) |  |  |
|  | *Patient-related** | *34 (13)* | Characteristics: basic digital affinity or experience in using digital applications, good language skills, younger age, a higher level of functionality, certain level of frustration tolerance in the face of technical problems, general openness to new technologies  Clinical aspects: Patients without an acute crisis, day clinic contexts involving more independent patients and longer stays, an increased willingness to change, recognisable therapeutic added value for patients  Other factors: stable, trusting therapeutic relationships | ”*Well, a younger age […] is a certain pull factor, so to speak. And there is perhaps more interest. And of course a certain affinity for technology on the patient side. Of course, the general motivation for therapy, right? In other words, how much do I want to engage with the treatment outside of the therapy services? So the higher the motivation for therapy and change, the higher, I think, the willingness to use Curamenta as an additional service.” (Interview 8 [translated from German])* |
|  | *Professional-related** | *20 (13)* | Characteristics: own digital affinity, openness to new things  Working conditions: availability in terms of time (for use in everyday clinical practice, as well as for familiarisation and training)  Added value: use of the platform perceived as beneficial, beneficial for the materials to be tailored closely to professionals' needs | *“Yes, that it really makes work easier. And that it's not an additional point of work. It makes you want to do it.” (Interview 4 [translated from German]*). |
|  | *Structural** | *19 (10)* | Support: support from superiors, support from central staff units, individuals responsible for the platform should actively support it and demonstrate their commitment to its implementation;  Structure: clear timetables and defined processes for introducing the platform to various clinical areas; synergies should be exploited through joint events, such as central onboarding events for all newly admitted patients.  Training: training courses (including introductory and further training days), for as many employees as possible, across all professional groups  Visibility: increased presence of the platform in cross-clinic exchanges (e.g. meetings of assistant doctors or therapeutic specialist teams) | *“That means making it easier if the medical director communicates very clearly, especially on the treatment side, […] And there will be very clear framework conditions/that is, a tighter corset on how it is to be used for which patient. […] for example, will no longer be given in paper form, if patients want it, they have to register, then they get it digitally. So if there's a bit more stringency in there.” (Interview 11 [translated from German])* |
|  | *Organisational** | *29 (15)* | Aim: having a shared understanding of the objectives and use of the platform within the team; agreements on the required materials, processes and responsibilities beforehand  Integration: Integrating the platform into existing routines, such as morning meetings, patient rounds and team meetings; desire for teams to work across sectors, for example in pre- and post-inpatient care | *“I think that fits in with the fact that team structures can really be addressed and not individuals. Yes, where a vacation or sickness replacement alone would be incredibly difficult to organize from my point of view. […]. That you can create team structures or organizations where someone from the team can look in specifically and then really answer the information or pass it on, coordinate it.” (Interview 4 [translated from German])* |
|  | *Technical** | *27 (11)* | Connection and equipment: a sufficient and free supply of Wi-Fi and suitable end devices for patients and professionals  App: professional app, automated feedback and push notifications from the platform for patients and professionals  Integration: improved interfaces with the hospital information system  Support: on-site technical support within the clinic | *“So basically, a well-functioning app makes this easier. It has to work on a cell phone or iPad. Or something like that that the patient has. And that's what the patient expects today. They do everything with it. So they do their banking with it, [...] buy their products with it. And in principle, a system like this must also work on an iPhone or a cell phone. Like this. And it has to be inviting. And that it is simple and intuitive.”* *(Interview 9 [translated from German])* |
|  | *other* | *6 (6)* |  |  |
| Barriers for implementation* | | 157 (15) |  |  |
|  | *Patient-related** | *34 (15)* | Clinical aspects: type of disorder (e.g. severe psychosis, media addiction or an acute event), concentration problems, difficulty structuring oneself, the short length of stay  Patient characteristics: or a lack of previous IT experience, advanced age of patients  Other: patients' general rejection of the platform | *“And then in an area where the patients are at an age where this is simply not yet so commonplace. So I don't know, from sixty, fifty, they don't necessarily have to be suffering from dementia to be treated by us. I think you'd probably have to look first, okay, do they have the necessary technical knowledge to be able to deal with it well?” (Interview 15 [translated from German])* |
|  | *Professional-related** | *30 (12)* | Characteristics: a lack of affinity with technology  Workload: additional time burden, high workload and tedious introduction of colleagues  Clinical aspects: fear of being overwhelmed due to open communication channels with patients, unclear responsibilities in the event of illness  Conviction: low motivation due to a lack of relevant or suitable content on the platform; a lack of perceived added value of the platform for professionals' own work | *“Yes, it's always an extra. So it's not as if you “save time” somewhere else. It's quite clear that it has to be packed into the daily work routine somehow, where it perhaps doesn't fit in at all.” (Interview 4 [translated from German])* |
|  | *Structural** | *27 (12)* | Integration: failure to integrate the platform into existing processes; the platform was not fully introduced in the clinics, meaning that some patients had access while others did not, which was perceived as unfair  Personnel: The scarcity of personnel and limited time resources in everyday clinical practice ( incl. staff rotation, personnel bottlenecks or high workloads); Concerns about data protection and the general experience that many digitalisation projects in the past were not pursued in the long term led to a wait-and-see attitude among professionals.  Hardware: a lack of hardware for professionals  Training: training courses that were held too long ago were also cited as barriers. | *“Ultimately, our biggest problems are that everything from data protection to fire safety takes priority. And in a hospital structure we [...] suffocate in data protection. And that of course hinders the whole thing, not technically, but in terms of content. The fact that we have a digital infrastructure that is basically from the digital Stone Age. This applies to many hospitals, and we are no exception.”* *(Interview 13 [translated from German])* . |
|  | *Organisational** | *21 (13)* | Personnel: bottlenecks and a lack of time and resources (e.g. for inductions and additional meetings); not all team members had received training, staff turnover, unclear allocation of responsibilities within the team, a lack of regular reminders about the platform in everyday working life.  Usage: the lack of shared use of logins by medical and psychological staff  Conviction: a lack of conviction about the benefits of the platform also led to uncertainty within the teams.  Experiences: Negative experiences with non-functioning technology had a demotivating effect on the teams. | “*Well, I think that's the biggest organizational obstacle that you have to somehow fit it into your daily routine. […] I think maybe also because you don't get a notification when there's something new from patients in Curamenta. At least that's not what I get at the moment.” (Interview 15 [translated from German])* |
|  | *Technical** | *29 (12)* | Connection and devices: lack of internet reception on the hospital premises, missing or inadequate end devices for patients  App: difficulties logging in, a lack of app compatibility for some modules, lengthy data protection and approval processes  Interfaces: repeated failures of the interfaces with hospital information systems (HIS). | *“Other aspects would be, for example, the problem of, yes, the interface between the HIS and Curamenta, which is repeatedly disrupted and therefore also greatly reduces the previously mentioned added value. But also, precisely, the aspects that we have already mentioned, for example extensive data protection, extensive approvals, naturally provide a strong obstacle in the general process.” (Interview 1 [translated from German])* |
|  | *other* | *11 (5)* |  |  |

No. = Number

| Category | | Codings (No. of persons out of 15) |
| --- | --- | --- |
| General experiences of non-users and users* | | 6 (5) |
| Reasons for non-usage* | | 7 (5) |
| Description platform usage in clinical care* | | 38 (10) |
| User-friendliness for patients* | | 48 (12) |
| Experience of professionals * | | 47 *(13)* |
|  | *Effects on everyday working life** | *11 (6)* |
|  | *Technical implementation** | *20 (10)* |
|  | *other* | *16 (11)* |
| Advantages of usage* | | 43 (15) |
|  | *For clinical care** | *19 (10)* |
|  | *For professionals ** | *4 (2)* |
|  | *For patients ** | *7 (5)* |
|  | *Economical use of ressources*** | *3 (2)* |
|  | *Other* | *10 (7)* |
| Disadvantages of usage* | | 32 (14) |
|  | *For clinical care** | *9 (5)* |
|  | *For patients** | *4 (4)* |
|  | *For professionals ** | *6 (5)* |
|  | *Technical aspects** | *3 (2)* |
|  | *other* | *10 (8)* |
| Acceptance* | | 60 (15) |
|  | *Relevance of patient portals** | *25 (14)* |
|  | *Recommendation of Curamenta by professionals** | *15 (15)* |
|  | *Promoting acceptance*** | *20 (11)* |
| Requirements for implementing Curamenta in routine clinical care* | | 40 (15) |
|  | *Structural aspects** | *18 (9)* |
|  | *Organisational aspects** | *2 (2)* |
|  | *Technical aspects** | *7 (5)* |
|  | *other* | *13 (11)* |
| Desired adjustments to the platform* | | 72 (15) |
|  | *For professionals** | *27 (10)* |
|  | *For patients** | *30 (14)* |
|  | *other* | *15 (7)* |
| Further comments | | 10 (4) |

* = deductive category; ** = inductive category; No. = number

1. **Expert consensus**

6.1 Overview on recommendations for implementation

* recommendation from first round: was split into 5a and 5b: ** new recommendations from second round; N= total number; n = number of ratings obtained; M = mean; SD = Standard deviation.

6.2 Average expert rating of all recommendations regarding importance of each recommendation (RA)


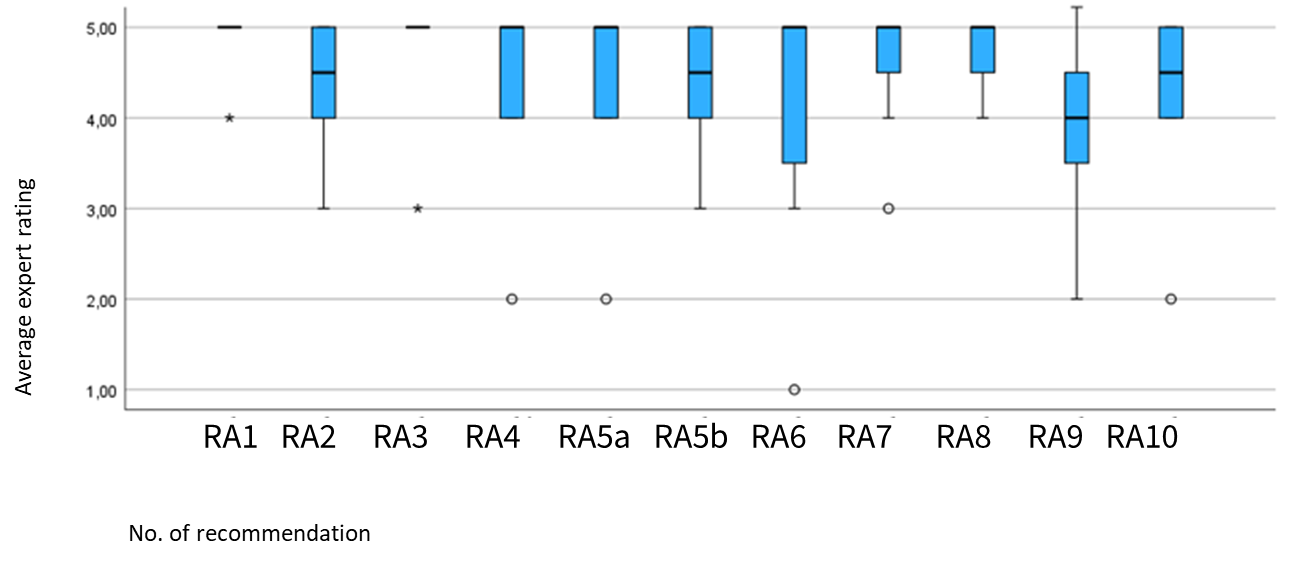


6.3 Rating of benefits for patients and professionals of selected recommendations by expert group (% of rating 4 [high benefit] and 5 [very high benefit])

RA = Recommendation for action

6.4 Rating of feasibility in clinical routine (% of rating 1 [not at all feasible] and 2 [rather not feasible]) and effort (% of rating 1 [very high effort] and 2 [high effort]) of selected recommendations by expert

RA = Recommendation for action
