# Supplementary material for: Barriers and Facilitators for the Implementation of an Online Portal in Hospital Mental Health Care: Implementation Study
Source: JMIR Form Res. 2026 May 19;10:e82450. doi: 10.2196/82450 (PMC13186438; doi:10.2196/82450)
Supplement: Checklist 1 [file formative-v10-e82450-s002.docx]

**GRAMMS checklist (Good Reporting of a Mixed Methods Study) [22]**

| Reporting item | Where in manuskript |
| --- | --- |
| 1) Describe the justification for using a mixed methods approach to the research question | Page 5 |
| 2) Describe the design in terms of the purpose, priority and sequence of methods | Methods: This mixed-methods study was conducted with concurrent quantitative and qualitative approaches  Study design: page 7 and figure 1 |
| 3) Describe each method in terms of sampling, data collection and analysis | Methods:  Quantitative: page 7, 11,  Qualitative: page 7/8; 11  Delphi: pages 8/9; 11 |
| 4) Describe where integration has occurred, how it has occurred and who has participated in it | Figure 1 (page 7)  Recommendation development and expert consensus building on recommendation: page 21/22 |
| 5) Describe any limitation of one method associated with the present of the other method | Page 26, 28 |
| 6) Describe any insights gained from mixing or integrating methods | Pages 26, 28 |
