# Supplementary material for: Barriers and Facilitators for the Implementation of an Online Portal in Hospital Mental Health Care: Implementation Study
Source: JMIR Form Res. 2026 May 19;10:e82450. doi: 10.2196/82450 (PMC13186438; doi:10.2196/82450)
Supplement: Checklist 2 [file formative-v10-e82450-s003.docx]

**COREQ (Consolidated criteria for reporting qualitative studies): 32-item Checklist [23]**

| **Item-No.** | **Description** | **Page** |
| --- | --- | --- |
| **Domain 1: Research team and reflexivity** | | |
| 1 | Which author/s conducted the interview? | 10 |
| 2 | What qualifications did the researcher have? | 10 |
| 3 | What was their employment status at the time of the study? | 10 |
| 4 | Was the researcher male or female? | 10 |
| 5 | What experience or training did the researcher have? | - |
| 6 | Was a relationship in place before start of the study? | - |
| 7 | What did the participants know about the researcher? | - |
| 8 | Which characteristics were reported about the interviewer? | - |
| **Domain 2: Study design** | | |
| 9 | Which methodology was used for the evaluation? | 13 |
| 10 | How were participants selected? | 18 |
| 11 | How were participants recruited? | 10 |
| 12 | How many participants were interviewed for the study? | 18 |
| 13 | How many people refused to participate or dropped out? Reasons? | 18 |
| 14 | Where was the data collected? | 18 |
| 15 | Was anyone else present during the interviews besides the participants and researchers? | - |
| 16 | What are the important characteristics of the sample? | 18 |
| 17 | Were questions, prompts, guides provided by the authors? Was the interview pilot tested? | 10 |
| 18 | Were repeat interviews carried out? If yes, how many? | - |
| 19 | Did the researchers use audio or visual recording to collect the data? | 18 |
| 20 | Were field notes made during and/or after the interview? | - |
| 21 | How long did the interviews take? | 18 |
| 22 | Was data saturation discussed? | 19 |
| 23 | Were transcript returned to participants for comment and/or correction? | - |
| **Domain 3: Analysis and results** | | |
| 24 | How many researchers coded the data? | 13 |
| 25 | Did authors provide a description of the coding tree? | 19 |
| 26 | Were topics identified in advance or derived from the data? | 19 |
| 27 | What software was used for data analysis? | 13 |
| 28 | Did participants provide feedback on the results? | - |
| 29 | Were participant quotations presented to illustrate the topics/results? Was each quotation identified? | 19-21 |
| 30 | Was there consistency between the data presented and the results? | - |
| 31 | Were major topics clearly presented in the results? | 19-21 |
| 32 | Were minor topics presented and discussed? | - |
